# Role of Salivary MicroRNA as a Marker of Progesterone Resistance in Endometriosis: Preliminary Results from a Single-Institution Experience

**DOI:** 10.3390/biom15040493

**Published:** 2025-03-27

**Authors:** Matilde Degano, Giorgia Vesca, Michela Bulfoni, Silvia Zermano, Stefano Restaino, Martina Arcieri, Errico Zupi, Renato Seracchioli, Lorenza Driul, Daniela Cesselli, Giovanni Scambia, Anna Biasioli, Giuseppe Vizzielli

**Affiliations:** 1 Clinic of Obstetrics and Gynecology, Santa Maria della Misericordia University Hospital, Azienda Sanitaria Universitaria Friuli Centrale, 33100 Udine, Italy; matilde.degano@asufc.sanita.fvg.it (M.D.); silvia.zermano@asufc.sanita.fvg.it (S.Z.); stefano.restaino@asufc.sanita.fvg.it (S.R.); martina.arcieri@asufc.sanita.fvg.it (M.A.); lorenza.driul@asufc.sanita.fvg.it (L.D.); anna.biasioli@asufc.sanita.fvg.it (A.B.); 2 Institute of Pathology, Santa Maria della Misericordia University Hospital, Azienda Sanitaria Universitaria Friuli Centrale, 33100 Udine, Italy; vesca.giorgia@spes.uniud.it (G.V.); michela.bulfoni@asufc.sanita.fvg.it (M.B.); daniela.cesselli@asufc.sanita.fvg.it (D.C.); 3 PhD School in Biomedical Sciences, Gender Medicine, Child and Women Health, University of Sassari, 07100 Sassari, Italy; 4 Department of Molecular and Developmental Medicine, Obstetrics and Gynecological Clinic, University of Siena, 53100 Siena, Italy; errico.zupi@unisi.it; 5 Division of Gynaecology and Human Reproduction Physiopathology, IRCCS Azienda Ospedaliero—Universitaria di Bologna, Policlinico di Sant’Orsola, 40138 Bologna, Italy; renato.seracchioli@unibo.it; 6 DMED Department of Medicine, University of Udine, 33100 Udine, Italy; 7 Gynecologic Oncology Unit, Department of Woman, Child and Public Health, Fondazione Policlinico Universitario Agostino, Gemelli IRCCS, 00136 Rome, Italy; giovanni.scambia@policlinicogemelli.it

**Keywords:** miRNA, endometriosis, progesterone resistance, biomarkers, prognosis

## Abstract

This feasibility study explores the potential of salivary microRNAs (miRNAs) as non-invasive biomarkers for diagnosing endometriosis and assessing treatment response. Almost one-third of patients with endometriosis do not respond to the standard progestin treatment due to various mechanisms of progesterone resistance. MiRNAs, recognized for their stability in body fluids and role in gene regulation, may offer new diagnostic and prognostic opportunities as they are involved in the pathogenic pathways of endometriosis and progesterone resistance. We sequenced salivary miRNAs in three cohorts of patients: control women without endometriosis and patients with endometriosis who responded and did not respond to standard progestin treatment. This aims to identify the differential miRNA expression profiles associated with therapeutic response to dienogest. The preliminary results demonstrate the feasibility of miRNA sequencing from saliva and reveal distinct miRNA profiles between responders, non-responders, and controls. Key miRNAs, including mir-3168, the mir-200a family, and mir-93-5p, emerged as potential biomarkers, showing significant differential expression linked to both endometriosis presence and treatment response. Further validation of these findings in larger cohorts could pave the way for miRNA-based diagnostic and prognostic tools, potentially reducing diagnostic delays and personalizing treatment approaches for endometriosis patients, also with new target therapies.

## 1. Introduction

The complex heterogeneity of endometriosis pathogenesis, histology, and clinics often makes the diagnostic process lengthy and challenging, with an 8- to 12-year delay between symptom onset and diagnosis [1]. Recent scientific literature has focused on developing new non-invasive diagnostic tools to identify endometriosis more rapidly and earlier, reducing diagnostic delays and enabling prompt treatment initiation [2]. Moreover, biomarkers can potentially monitor the progression of the disease, assess the recurrence risk, and enable secondary prevention strategies. This could expand treatment options and inform timely decisions, particularly regarding fertility [3]. Numerous potential biomarkers have been proposed over time, but insufficient or low-quality evidence currently limits their clinical application [1]. Among these, microRNAs (miRNAs) have emerged as a promising class of nucleic acid-based biomarkers, supported by evidence of their relevance in other diseases, including various cancers and degenerative disorders [3,4,5,6].

Many of the genes regulated by miRNAs are known to be involved in crucial processes in the pathogenesis of endometriosis [7,8,9,10,11,12]. The most recent literature [3] concludes that a panel of miRNAs may constitute a robust biomarker to diagnose the disease with sufficient sensitivity and specificity. MiRNAs were analyzed in multiple samples (blood, urine, and serum) [13], but saliva [2] was found to be an excellent substrate for miRNA detection, with a higher concentration of isolated miRNAs than plasma, serum, and urine, comparable stability and reproducibility, and greater feasibility in collecting the sample [3,14].

MiRNAs appear to also be involved at multiple levels in progesterone resistance. The use of progestins and combined oral contraceptives is the first-line treatment for endometriosis [1], particularly for long-term management. Despite the widely proven benefit of progestin therapies, it is estimated that they are ineffective inone- third of symptomatic patients [15,16,17]. The inability of the endometrium to respond adequately to progesterone is known as progesterone resistance [18]. The loss of progesterone efficacy is a multifactorial process [18,19,20,21]. The main mechanism involves the reduced expression of progesterone receptors (PRs) in endometriosis patients [22,23], especially affecting PR-B [18,24]. Several miRNAs are associated with progesterone resistance by modulating the expression of PRs [25,26,27] and influencing other dysregulated gene networks [28,29,30,31].

However, despite recent advancements, no predictive biomarkers for responses to medical therapy in endometriosis have been identified, even among miRNAs. The most recent guidelines emphasize the need to identify prognostic markers in endometriosis to help determine which patients are more likely to respond to specific treatments, given the lengthy history of therapies and surgeries these patients often undergo.

This study aims to investigate the potential role of salivary miRNAs in the diagnosis of endometriosis and in the early assessment of responses to standard therapy and progesterone resistance.

## 2. Materials and Methods

### 2.1. Objectives and Study Design

The preliminary results of a prospective, single-institution observational feasibility study are presented. The primary objective of this study was to determine the feasibility of sequencing miRNAs from saliva samples. The secondary objective was to assess the differential expression profile of salivary miRNAs between patients who responded to standard medical treatment with progestin and those who did not, as well as between patients diagnosed with endometriosis and a non-endometriosis population.

This study involved patients diagnosed with endometriosis attending the Obstetrics and Gynecology Clinic at Santa Maria della Misericordia Hospital in Udine who had not yet begun medical treatment, as well as control healthy subjects without an endometriosis diagnosis attending the same clinic. The inclusion criteria were age ≥18 years, reproductive age, clinical and ultrasound [32] or histological diagnosis of endometriosis, and signed informed consent. The following exclusion criteria were also applied: pregnant patients; pre-menarchal or post-menopausal status; Chronic Pelvic Pain Syndrome [33,34,35]; ongoing progestin therapy, BMI > 30 kg/m^2^; or a personal history of cancers, diabetes, coagulopathies, autoimmune diseases, or any other conditions that may affect the measurement of salivary miRNA related to endometriosis.

The Institutional Review Board of the Medicine Department of the University of Udine (IRB-DMED) approved the study with protocol No. 106/2024 (8 April 2024).

Sixteen patients with endometriosis and twelve healthy controls were enrolled between April and July 2024, and the following data were collected for each patient: age, parity, menstrual history, BMI, endometriosis-related symptoms, pain history including pain severity measured using the numerical rating scale (NRS) [36], comorbidities, current and previous therapies, prior surgeries, endometriosis phenotype, and imaging (ultrasound or MRI) results.

A saliva sample was collected before therapy for this initial pool of 28 patients. Subsequently, the 16 patients with endometriosis were prescribed continuous-regimen dienogest therapy at 2 mg daily, following international guidelines [1].

Four months after starting therapy, the patients with endometriosis were re-evaluated to assess symptom response. Treatment response was measured using the Patient’s Global Impression of Change (PGIC) [37,38,39], a seven-point, single-item scale (from 1 = very significant improvement to 7 = very significant worsening) that patients are invited to use to evaluate their overall condition since the start of treatment [37]. Patients reporting a score of 1 or 2 were identified as responders, while those scoring ≥ 3 were classified as non-responders [37].

The first sequencing of salivary samples was performed on 12 patients: 4 controls and 8 patients with endometriosis, including 4 responders and 4 non-responders.

The relevant data were recorded in a custom-designed electronic Excel database in anonymized form. The investigators and staff involved in the study adhered to the fundamental principles of good clinical practice.

### 2.2. Sample Collection, Processing, and Analysis

Each participant was provided with a sterile test tube containing a preserving solution (DNA/RNA Shield Safe Collection Kit, Zymo Research, Irvine, CA, USA) for saliva collection. The samples were stored according to the proper guidelines until the sequencing phase. The processing and analysis were performed as follows:

1. *Nucleic Acid Extraction*

After initial centrifugation at 10,000× *g* for 20 min to remove cellular debris and contaminants, RNA was extracted using the miRNeasy Advanced Micro Kit (Qiagen, Hilden, Germany), following the manufacturer’s instructions. The final yield was determined through fluorimetry using the Qubit RNA HS (High Sensitivity) Assay on the Qubit Fluorometer 3.0 (Invitrogen Co., Waltham, MA, USA). The extracted RNA was stored at −80 °C until analysis.

2. *NGS Library Preparation*

MiRNA libraries were prepared using the QIAseq microRNA Library Kit (Qiagen, Hilden, Germany). Adaptors were ligated to the RNA, followed by reverse transcription to generate cDNA. After cDNA synthesis, each library was amplified using unique dual indexes (QIAseq UX Index—Qiagen, Hilden, Germany) (PCR). The libraries were quantified using the Qubit 2.0 (Invitrogen, Waltham, MA, USA) with the HS dsDNA Assay Kit (High Sensitivity), and fragment sizes (170 bp) were verified on the Bioanalyzer 2000 (Agilent, Santa Clara, CA, USA). The individual libraries were pooled into a single pool and sequenced in 74 bp paired-end mode using Illumina sequencing technology.

3. *Bioinformatics Analysis*

FASTQ files were uploaded to the RNA-seq Analysis Portal (RAP; GeneGlobe, Qiagen, Hilden, Germany) to perform primary, secondary, and tertiary analyses of sequencing data. Briefly, the raw data were first demultiplexed, and the sequences were then mapped to the reference genome (miRbase) [40]. Differential miRNA expression between patient groups was calculated using the DESeq2 algorithm, with a significance threshold of False Discovery Rate (FDR) adjusted *p*-value < 0.01. For the functional characterization of differentially expressed miRNAs, Ingenuity Pathway Analysis (IPA) and the Gprofiler2 R package (version 0.2.3) were used with default parameters, querying the Gene Ontology database [41]. A significance Z-score threshold of <−2 or >+2 was applied to highlight the most relevant terms. Target genes of deregulated miRNAs were retrieved from TarBase [42].

### 2.3. Statistical Analysis

Quantitative variables were summarized using appropriate descriptive statistics. Qualitative data were summarized using contingency tables. The normal distribution of quantitative variables was assessed using the Shapiro–Wilk test. Comparisons between groups were made using the *t*-test or Mann–Whitney U-test, depending on the results of the Shapiro–Wilk test. The χ^2^ test was used to analyze categorical values; Fisher’s exact test was employed when the assumptions for the χ^2^ test were not met. The association between quantitative variables was explored using Pearson correlation and Spearman correlation analyses.

## 3. Results

### 3.1. Baseline Characteristics and Treatment Response

The baseline characteristics of the 12 patients enrolled are reported in Table 1. The three populations were found to be homogeneous regarding their baseline characteristics. However, since the reported data are preliminary and the groups are small in number, all the statistical calculations performed are underpowered. Regarding symptoms and the presence of different endometriosis phenotypes, only responder and non-responder patients were compared, assuming that the control group was asymptomatic and healthy. The two groups were homogeneous across all collected parameters, except for the reported NRS for dysmenorrhea. Data concerning dyspareunia are incomplete since one of the non-responder patients could not be evaluated in this aspect, as she had never had sexual intercourse.

**Figure 1:**
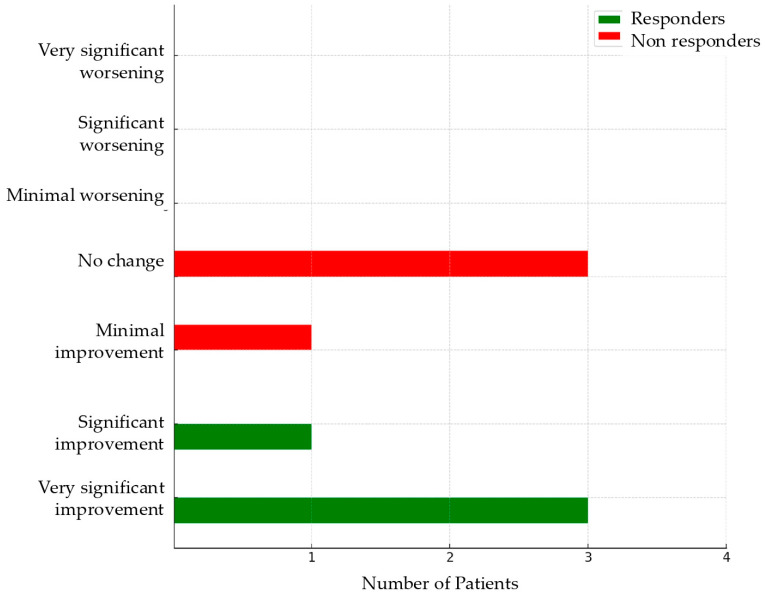
Patient’s Global Impression of Change (PGIC) for responders vs. non-responders.

No patients indicated a worsening of symptoms after four months of therapy, with the maximum score on the PGIC being 4 (Figure 1), which corresponds to no substantial change. Among the non-responders, three patients reported a score of 4 and one patient scored 3. In contrast, three responders reported the highest score (very significant improvement) and one scored 2. The changes for each symptom in terms of the number of affected patients and NRS are detailed in Table 2.

### 3.2. miRNA’s Principal Component Analysis

**Figure 2:**
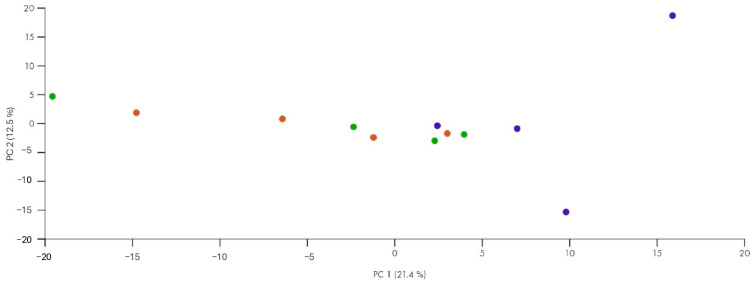
Principal Component Analysis (PCA) of miRNAs differentially expressed in the three groups (green = non-responders, blue = controls, and red = responders). PCA was performed using as predictors all the miRNAs identified as differentially expressed in at least one patient group in the comparison of each patient group to the control state.

A PCA (Principal Component Analysis) was then performed to visualize the variation in miRNA expression between the groups. The first PCA plot (Figure 2) reveals good separation between the groups but with some overlap between the patients responding and those not responding to therapy. This suggests that certain miRNAs may be differentially expressed between controls, responders, and non-responders. Although some overlap between the endometriosis subgroups is observed, the controls appear more clustered, indicating relatively homogeneous miRNA expression compared to the endometriosis groups.

**Figure 3:**
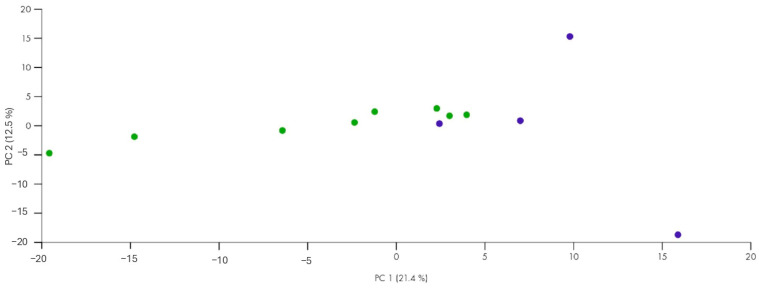
PCA plot of the controls (blue) vs. all patients with endometriosis (responders and non-responders) (green).

In the second PCA plot (Figure 3), only the control group and the endometriosis patients are represented, with no distinction between responders and non-responders. The analysis suggests that while the control group exhibits tighter clustering, indicating consistent expression profiles, the endometriosis group is more dispersed, reflecting more significant variability in miRNA expression. This may reflect the biological complexity of the condition and the differences between responders and non-responders to therapy. Still, there seems to be a tendency toward some distinction, suggesting that certain miRNAs may differentiate the groups with and without endometriosis.

### 3.3. Differential miRNA Expression Between Responders and Non-Responders

**Figure 4:**
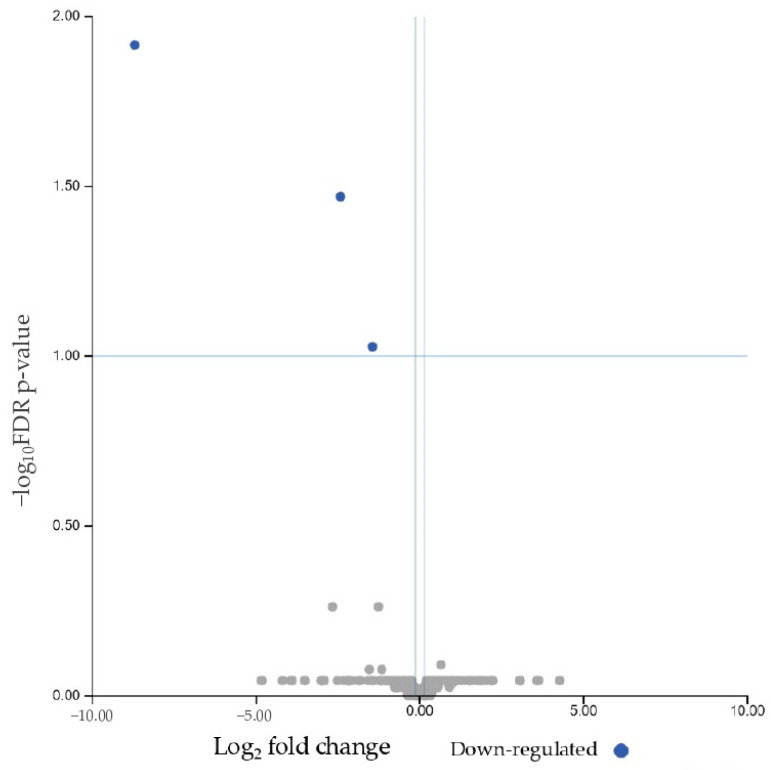
Volcano plot showing the relationship between fold change and statistical significance (FDR-adjusted *p*-value) of miRNAs differentially expressed in non-responder patients compared to responders. The blue points in the plot represent the significantly (FDR ≤ 0.1) downregulated miRNAs (see Table 3).

The next step in the analysis was to identify the specific miRNAs that were differentially expressed between the three groups. A preliminary evaluation of the differential expression of miRNAs between responder and non-responder patients was performed. Considering an FDR-adjusted *p* value ≤ 0.1, three miRNAs were found to be downregulated in non-responders compared to responders: let7c-5p, mir200a-3p, and mir3168 (Table 3 and Figure 4).

**Figure 5:**
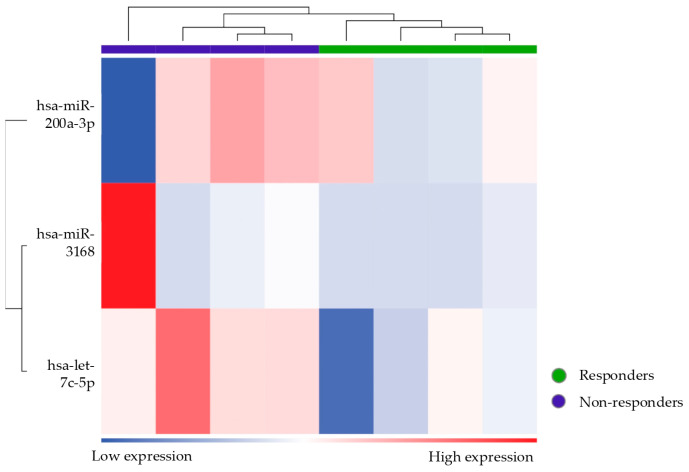
Heatmap showing the unsupervised clustering of samples based on the expression levels of the three differentially expressed miRNAs (let7c-5p, mir200a-3p, and mir3168) in responder and non-responder patients. The color scale represents the level of upregulation (red) and downregulation (blue) of miRNA. The clustering based on the three miRNAs effectively separates responders (purple bar) from non-responder patients (green bar).

The differential expression of these three miRNAs allows for the homogeneous clustering of the two groups. The heatmap (Figure 5) illustrates the expression level of the three miRNAs across responder and non-responder patients and shows a clear separation between them, with distinct clustering patterns for each group. Functional analysis of the pathways affected by the identified miRNAs highlights their involvement in the Nucleosome Assembly Protein 1-Like 1 (NAP1L1) Transcription Regulation Signaling Pathway.

**Figure 6:**
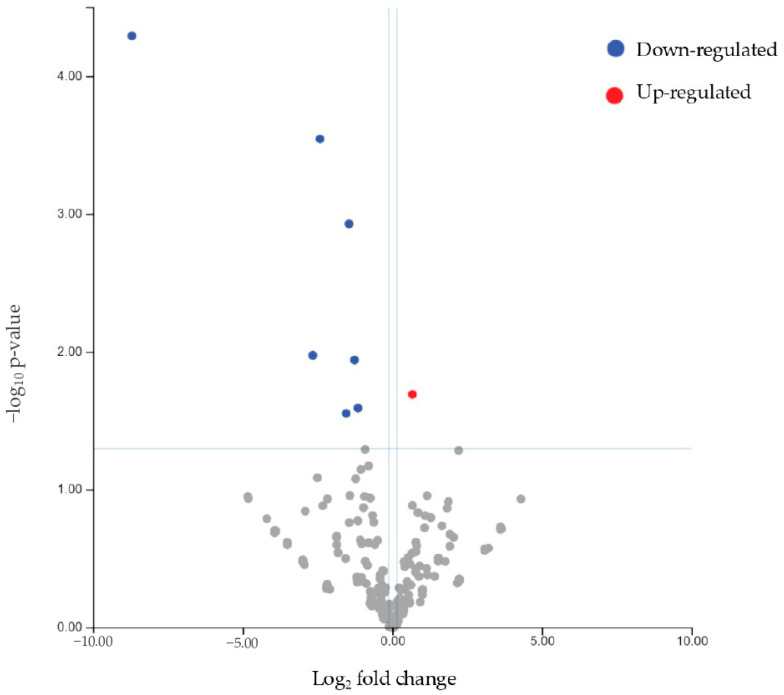
Volcano plot showing the relationship between fold change and statistical significance (*p*-value) of miRNAs differentially expressed in non-responder patients compared to responders. The blue and red points in the plot represent the significantly (*p*-value ≤ 0.05) downregulated and upregulated miRNAs, respectively (see Table 4).

**Figure 7:**
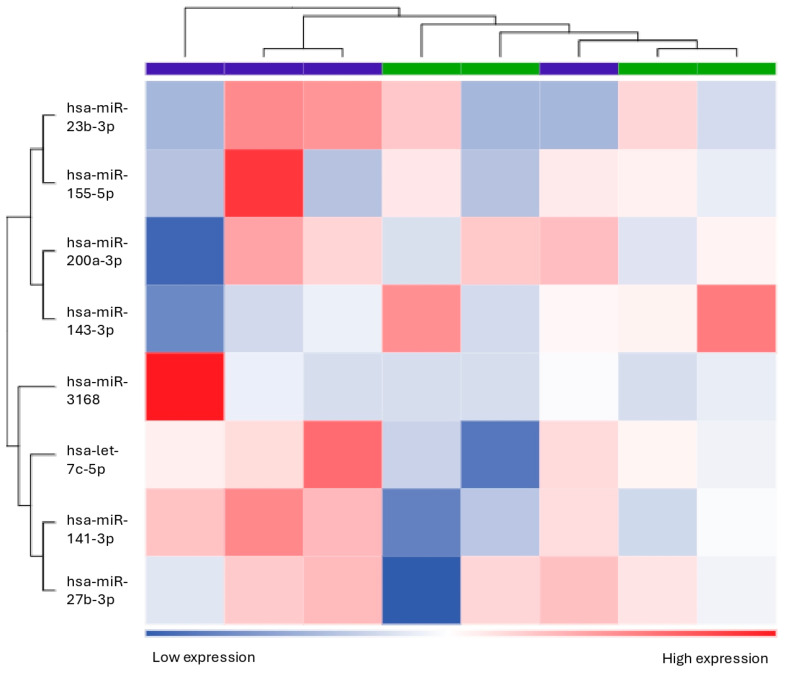
Heatmap showing the unsupervised clustering of samples based on the expression levels of the eight differentially expressed miRNAs (let7c-5p, mir200a-3p, mir3168, mir23b-3p, mir27b-3p, mir141-3p, mir155-5p, and mir143-3p) in responder and non-responder patients. The clustering based on the eight miRNAs was effective in discriminating responders (purple bar) from non-responders (green bar) in all cases except one non-responder.

When considering differentially expressed miRNAs with a *p*-value ≤ 0.05 (instead of the more stringent FDR value) in non-responders compared to responders, let7c-5p, mir200a-3p, mir3168, mir23b-3p, mir27b-3p, mir141-3p, and mir155-5p were found to be downregulated, while mir143-3p was found to be upregulated (Table 4, Figure 6). However, the identified targeted pathway of the eight differentially expressed miRNA remained the same (NAP1L1). Figure 7 displays the heatmap related to the unsupervised clustering analysis of the patients based on this second dataset. While most non-responders clustered together, showing consistent distinct patterns from the responders, one non-responder was incorrectly classified as a responder.

### 3.4. Differential miRNA Expression Between Patients and Controls

**Figure 8:**
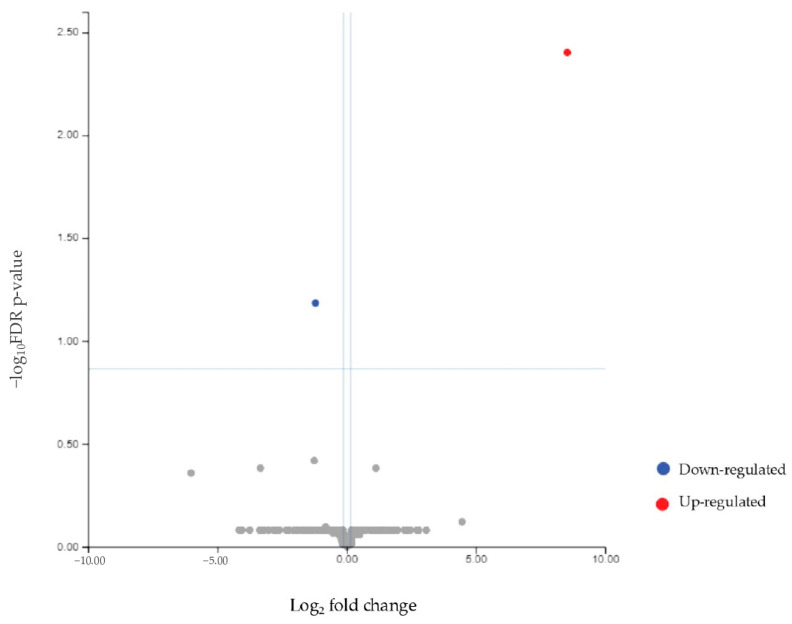
Volcano plot showing the relationship between fold change and statistical significance (FDR-adjusted *p*-value) of miRNAs differentially expressed in patients compared to healthy controls. The blue and red points in the plot represent the significantly (FDR ≤ 1) downregulated and upregulated miRNAs, respectively (see Table 5).

**Figure 9:**
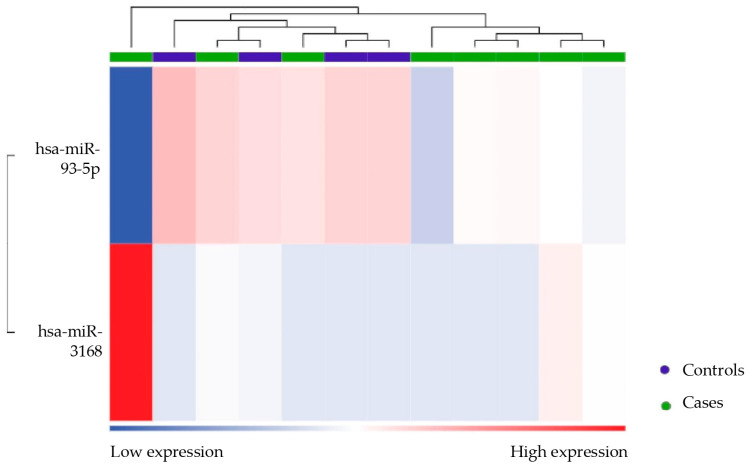
Heatmap showing the unsupervised clustering of samples based on the expression levels of differentially expressed miRNAs (mir93-5p and mir3168) in patients (green bar) compared to controls (purple bar). A group of patients clustered together with healthy subjects.

Secondly, miRNA differentially expressed between patients (both responders and non-responders) and healthy controls was evaluated. Considering an FDR value ≤ 0.1, two differential miRNAs emerged: mir93-5p was downregulated, while mir3168 was upregulated (Table 5 and Figure 8). The heatmap (Figure 9) demonstrates the clustering of the case and control groups, further supporting their distinction. The difference in clustering patterns between Figure 7 and Figure 9 highlights the variability in miRNA expression within the endometriosis population compared to the more uniform profiles seen when distinguishing patients from healthy controls.

**Figure 10:**
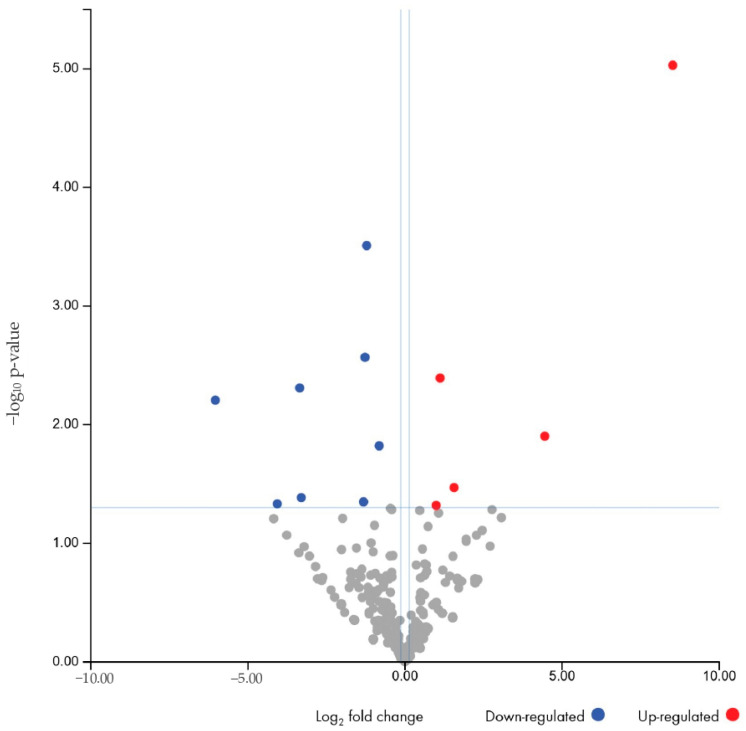
Volcano plot of miRNAs differentially expressed in patients compared to controls with a *p*-value ≤ 0.05.

**Figure 11:**
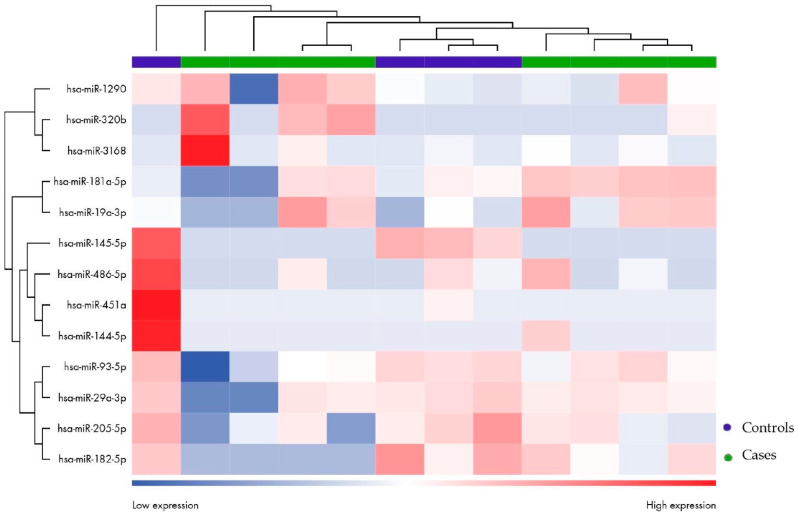
Heatmap showing unsupervised clustering of samples based on the expression levels of differentially expressed miRNAs in patients (green) compared to controls (purple).

When considering differentially expressed miRNAs with a *p*-value ≤ 0.05, hsa-miR-3168, hsa-miR-181a-5p, hsa-miR-320b, hsa-miR-19a-3p, and hsa-miR-1290 were found to be upregulated. Hsa-miR-93-5p, hsa-miR-205-5p, hsa-miR-486-5p, hsa-miR-451a, hsa-miR-29a-3p, hsa-miR-144-5p, hsa-miR-182-5p, and hsa-miR-145-5p were found to be downregulated (Table 6 and Figure 10 and Figure 11).

### 3.5. Differential miRNA Expression Between Responders and Controls

**Figure 12:**
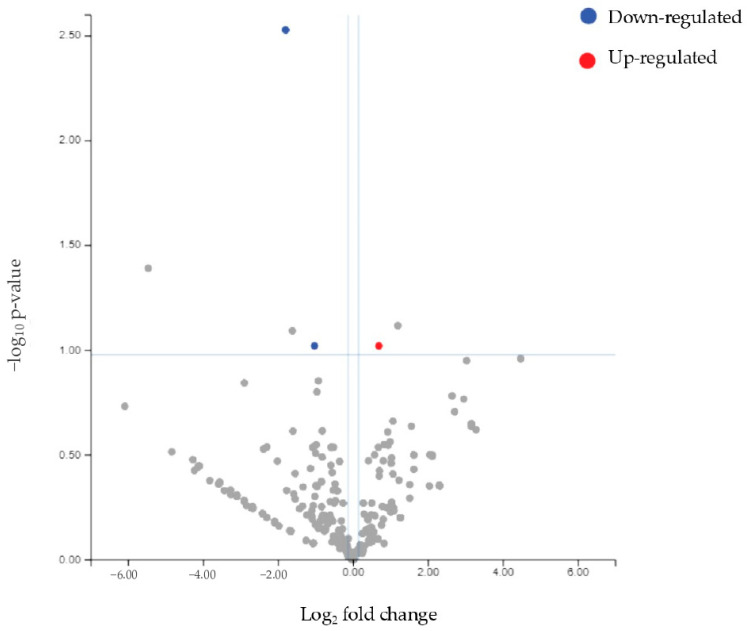
Volcano plot of miRNAs differentially expressed in responders compared to controls.

**Figure 13:**
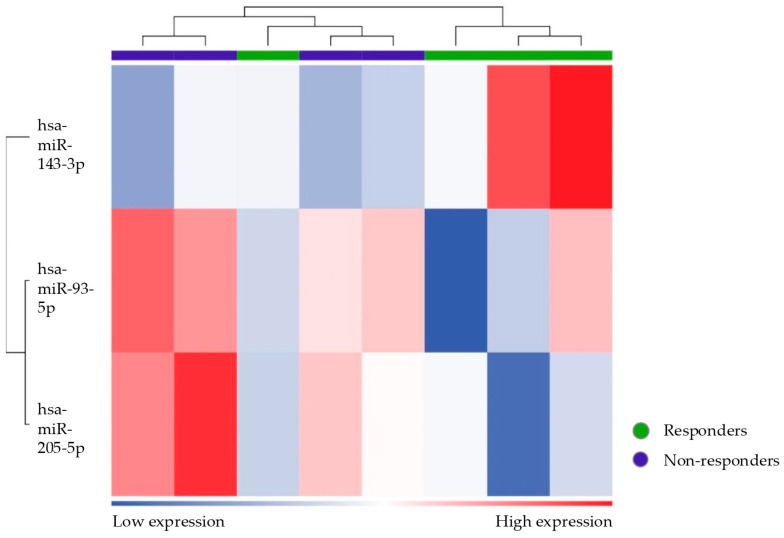
Heatmap showing unsupervised clustering of samples based on the expression levels of differentially expressed miRNAs (mir93-5p, mir205-5p, and mir143-3p) in responders (green) compared to controls (purple).

The differential miRNA expression between responder patients and controls was also evaluated. Three differential miRNAs emerged: mir93-5p and mir205-5p were downregulated, while mir143-3p was upregulated (FDR value ≤ 0.1) (Table 7 and Figure 12 and Figure 13). The clustering analysis reveals a distinct separation between the two groups.

### 3.6. Differential miRNA Expression Between Non-Responders and Controls

Finally, the differential miRNA expression between non-responder patients and controls was evaluated. The two differential miRNAs previously identified between cases and controls were confirmed: mir93-5p was downregulated and mir3168 was upregulated in non-responder patients compared to controls (FDR value ≤ 0.1).

## 4. Discussion

First, this study demonstrated that it is feasible to sequence miRNAs from saliva, confirming data from the literature [2].

When examining the differential miRNA expression between patients (responders and non-responders) and controls, we identified mir93-5p as significantly downregulated and mir3168 as upregulated. These findings suggest that these miRNAs could serve as critical biomarkers for the presence of endometriosis. Interestingly, mir3168 was also significantly downregulated in non-responders compared to responders, highlighting its potential role in modulating the therapeutic response in endometriosis. This miRNA was found to be significantly downregulated in blood samples in a previous French study [44], which compared endometriosis patients to controls. This finding is noteworthy as it demonstrates dysregulation of the same miRNA across different biological sample types. miR-3168 is not as well-characterized as other miRNAs, but some studies have associated miR-3168 with the regulation of cell proliferation and tumor progression, indicating its potential role in oncogenesis [45]. However, as of now, its role in endometriosis still requires further investigation.

miR-93-5p was upregulated in healthy controls compared to patients (either considering the subgroup of responders or non-responders separately). In the literature, miR-93 (the precursor of miR-93-5p) has been shown to target Matrix Metalloproteinase 3 (MMP3) and Vascular Endothelial Growth Factor A (VEGF-A), which are involved in endometriosis by contributing to tissue remodeling, angiogenesis, and the proliferation of endometriotic lesions [46]. The deregulation of miR-93 is believed to contribute to endometriosis by upregulating MMP3 and VEGFA, suggesting potential therapeutic targets. This result is in contrast with another study [47] in which miR-93 was demonstrated to be upregulated in the eutopic endometrium of women with endometriosis when compared to the controls. Such a difference between results is likely due to the different tissues analyzed in the comparison. Moreover, in this study, the expression of miR-93 was different between superficial and deep lesions, with greater expression in the latter.

Furthermore, miR-93-5p has been identified through in silico analysis as a negative regulator of DROSHA, a crucial protein for miRNA production whose alterations can affect gene regulation and have been implicated in various diseases, including endometriosis [48].

A further study investigating, through bioinformatics analysis, the regulatory networks between transcription factors (TFs), miRNAs, and genes in the pathogenesis of ovarian endometriomas identified the RELA–miR-93-5p–HIF1A axis as potentially involved in the development of this condition [49]. However, in this study, the highest degree of intersection among nodes in the interaction networks between TFs, miRNAs, and hub genes was observed for miR-155-5p, within the YY1–miR-155-5p–HIF1A axis. The study hypothesized that these regulators play a role in the pathogenesis of ovarian endometriomas: transcription factors negatively regulate the expression of miRNAs, whose reduction leads to the loss of their repressive action on downstream target genes, resulting in their increased expression and the activation of various pathways, including the HIF-1 signaling pathway, glycolysis, and ferroptosis [49].

This study’s analyses also revealed intriguing data on miR-143-3p, showing an increasing gradient in its expression: it was upregulated in non-responder patients compared to responders and in responders compared to controls. Conflicting results are found in the literature, showing that this miRNA is either upregulated or downregulated in patients with endometriosis [9,47,50,51,52,53]. However, functional studies suggest that miR-143-3p directly targets Vasohibin 1 (VASH1), activating TGF-β1 signaling and promoting cell migration and invasion in endometriosis. Increased expression of TGF-β1 contributes to the downregulation of PR expression in women with endometriosis [54]. These findings suggest that the dysregulation of miR-143-3p may lead to progesterone resistance and increased progression of endometriotic lesions [31].

Among the responder vs. non-responder patients, the miRNAs let-7c-5p, mir-200a-3p, and mir3168 were found to be differentially expressed.

Members of the let-7 family, including let-7c, are found to be downregulated in endometriosis. Reduced levels of let-7 miRNAs are thought to promote the survival and proliferation of endometriotic cells, contributing to disease progression, as it fails to suppress oncogenic and inflammatory pathways that let-7 typically targets [55]. Moreover, the let-7 miRNA has been studied in breast cancer as strongly related to the induction of PR-B via activation of the E2 binding factor 1 (E2F1) promoter [56]. If let-7 also induces PR-B in endometriosis, its downregulation in endometriosis patients would lead to an environment with suppressed PRs, particularly affecting PR-B, contributing to progesterone resistance [18].

miR-200a-3p is part of the miR-200 family, which has been linked to the regulation of the epithelial-to-mesenchymal transition (EMT), an essential process for the invasiveness of endometrial cells. Studies have suggested that the dysregulation of miR-200a-3p in endometriosis could enhance the migratory and invasive potential of endometrial stromal cells, contributing to lesion formation and progression. The miR-200 family may also be involved in modulating progesterone signaling through PGRMC (membrane-associated progesterone receptor component), further contributing to the development of progesterone resistance [29].

Additional references from the literature regarding the miRNAs found to be differentially expressed in this study are reported in Table 8.

The NAP1L1 Transcription Regulation Signaling Pathway was identified as a key pathway associated with the differentially expressed miRNAs and confirmed in two of the conducted analyses. This gene is involved in cell proliferation and has been implicated in the pathogenesis of various carcinomas [66,67]. However, its role in endometriosis has not yet been described. Nonetheless, it is involved in the NF-κB signaling pathway, which is, in turn, implicated in the mechanism of progesterone resistance [17,68,69,70].

Another interesting finding is that miRNAs implicated in progesterone resistance could become therapeutic targets. Recent literature [71] demonstrated that metformin can regulate the expression of altered genes and miRNAs in faulty endometriotic mesenchymal stem cells with impaired differentiation, restoring their disrupted self-renewal/differentiation balance and making it a promising drug to reverse progesterone resistance and treat endometriosis. Even more surprising is that among the miRNAs involved in this study and restored by metformin, we find let-7 and miR-200.

Interestingly, one patient emerged as incorrectly classified according to unsupervised clustering, showing a miRNA expression profile that was more similar to that of responders despite being clinically categorized as a non-responder. Assuming there were no technical errors or issues with sampling, this anomaly could indicate underlying biological variability in microRNA expression patterns, reflecting a specific or transient condition that affects the response to therapy. Alternatively, it is possible that this patient was undergoing a transition phase toward a response to treatment, and this profile, similar to the responder ones, reflects an improvement in symptoms that have not yet been clinically detected. Another explanation could be that this patient has confounding factors (other than the exclusion criteria) influencing her miRNA expression profile. Moreover, by broadening the statistical criteria (from FDR to *p*-value) to define differentially expressed miRNAs, the ability to discriminate is lost. In fact, when using more stringent criteria, this patient does not emerge as an outlier.

The expression of miRNAs is dynamic and influenced by several factors such as age, ethnicity, physiological status, the presence of various diseases, smoking, and other external factors [3]. Therefore, choosing an appropriate control group is crucial when studying circulating miRNA expression. In selecting our patient groups, we aimed to make the populations as homogeneous as possible. However, one of the limitations of this study is that if healthy women are chosen as the control group, it is always possible that some of them may have asymptomatic endometriosis unless it is laparoscopically excluded. Similarly, when distinguishing between progesterone-resistant and non-resistant patients, we excluded any other potential causes of resistance, including chronic pelvic pain syndrome, primarily linked to central sensitization. However, many other confounding factors may exist, and a much larger population is undoubtedly needed to maximize the analysis.

In fact, another limitation of this study is the small sample size. However, it is intriguing that we found statistically significant results even with such small samples. It will be interesting to assess the final data from this feasibility study by analyzing the miRNAs in the other samples collected (currently underway) and later determine whether these identified miRNAs can be validated in a larger patient cohort using less costly techniques (real-time PCR).

A further limitation of our study actually concerns the lack of experimental validation of the miRNAs identified through sequencing. Confirming the differential expression of miRNAs by RT-qPCR, using a targeted approach like the TaqMan Advanced miRNA assay, would be a crucial step in strengthening our results. In future studies, it will be interesting to perform such validation on a new set of independent samples to individually assess the miRNAs identified in this study.

One of the main challenges of this study was accurately defining which patients were responding to therapy and which were not. The literature does not provide a unanimous definition for these two categories. Given that the primary goal of endometriosis treatment is to improve symptoms, this study selected a scale that reflects the patient’s subjective perception of treatment response: the Patient’s Global Impression of Change (PGIC) [38]. As in the study by Vercellini et al. [37], we included a score of 3 (= minimal improvement) in the non-response category because, in the author’s opinion and agreeing with more recent literature [37], a state of minimal improvement may not be considered sufficiently satisfactory to suggest continuing treatment for a patient with severe symptoms for which alternative options exist. The definition of a non-response was applied after 4 months of unsuccessful treatment. We considered this timeframe sufficient to define whether the patient responded to therapy. However, the literature lacks consensus regarding the timing of assessments [37].

## 5. Conclusions

Overall, these preliminary findings highlight the feasibility of using salivary miRNAs as non-invasive biomarkers for endometriosis, especially for treatment response. Differentially expressed miRNAs between responder and non-responder patients and between patients with and without endometriosis were found, some of which are consistent with the findings in the literature. Thus, these specific miRNAs appear to play roles in the dysregulation of PR signaling, contributing to progesterone resistance.

Developing an even deeper understanding of the mechanisms underlying the identified miRNAs could help in designing new strategies to improve patient response to therapies, restoring progesterone sensitivity, or developing alternative treatments bypassing resistance pathways. Furthermore, knowing a patient’s response to therapy in advance would allow her to be directed immediately toward other therapeutic strategies, such as surgery or ART.

This study, although promising, shows several limitations. The study sample is relatively small, and further validation in larger cohorts is required to confirm these findings. Additionally, longitudinal studies that follow patients over extended periods are essential to better understand how miRNA expression profiles evolve over time and in response to therapy.

In conclusion, this study identifies promising salivary miRNA candidates that may be biomarkers for diagnosing endometriosis and predicting treatment response. Future research should aim to refine these findings and explore the underlying mechanisms by which these miRNAs are involved in endometriosis pathophysiology and therapeutic outcomes.

## Figures and Tables

**Table 1:** Main characteristics of the included patients.

	Controls	Responders	Non-Responders	*p*-Value
Age (mean)	34.2	30.2	27.5	0.323
HMB *n* (%)	0	1 (25%)	1 (25%)	0.548
BMI (mean, Kg/m^2^)	21.7	19.5	24.6	0.145
Acyclic pain *n* (%)	0	2 (50%)	2 (50%)	1.0
Acyclic pain NRS (mean)	0	6	5.5	0.919
Dyspareunia *n* (%)	0	4 (100%)	2 (50%) *	0.536
Dyspareunia, Marinoff Dyspareunia Scale (Marinoff et al., 1992 [43]) ** (mean)	0	2.5	2	0.134
Dyschezia *n* (%)	0	2 (50%)	1 (25%)	0.536
Dyschezia NRS (mean)	0	6	4	0.355
Dysmenorrhea *n* (%)	0	4 (100%)	4 (100%)	1.0
Dysmenorrhea NRS (mean)	0	4.5	8.5	0.0001
Periovulatory pain *n* (%)	0	1 (25%)	1 (25%)	1.0
Periovulatory pain NRS (mean)	0	8	7	0.355
Endometriomas *n* (%)	0	2 (50%)	1 (25%)	0.536
DIE *n* (%)	0	2 (50%)	2 (50%)	1.0
Superficial endometriosis *n* (%)	0	1 (25%)	2 (50%)	0.536
Adenomyosis *n* (%)	0	1 (25%)	1 (25%)	1.0
Adhesion syndrome *n* (%)	0	2 (50%)	1 (25%)	0.536

* A patient could not be evaluated for dyspareunia as she had never engaged in sexual intercourse. ** The Marinoff Dyspareunia Scale describes the pain limitations to practice sexual intercourse: 0, no limitations in sexual intercourse; 1, causes discomfort but does not prevent sexual intercourse; 2, frequently prevents sexual intercourse; 3, completely prevents sexual intercourse.

**Table 2:** Symptoms and adverse effects in the two endometriosis groups at baseline (t0) and after 4 months of therapy (t4).

	Responders t0	Responders t4	Non-Responders t0	Non-Responders t4
Spotting *n* (%)	--	1 (25%)	--	2 (50%)
Acyclic pain *n* (%)	2 (50%)	0	2 (50%)	2 (50%)
Acyclic pain NRS (mean)	6	0	5.5	5.5
Dyspareunia *n* (%)	4 (100%)	3 (75%)	2 (50%) *	3 (75%) *
Dyspareunia, Marinoff Dyspareunia Scale41, ** (mean)	2.5	1.7	2	2
Dyschezia *n* (%)	2 (50%)	0	1 (25%)	1 (25%)
Dyschezia NRS (mean)	6	0	4	4
Dysmenorrhea *n* (%)	4 (100%)	1 (25%)	4 (100%)	4 (100%)
Dysmenorrhea NRS (mean)	4.5	1	8.5	6.75
Periovulatory pain *n* (%)	1 (25%)	0	1 (25%)	1 (25%)
Periovulatory pain NRS (mean)	8	0	7	5
Global Impression of Change (mean)	--	1.5	--	3.75

* A patient could not be evaluated for dyspareunia as she had never engaged in sexual intercourse. ** The Marinoff Dyspareunia Scale describes the pain limitations to practice sexual intercourse: 0, no limitations in sexual intercourse; 1, causes discomfort but does not prevent sexual intercourse; 2, frequently prevents sexual intercourse; 3, completely prevents sexual intercourse.

**Table 3:** Downregulated miRNAs in non-responders vs. responders.

miRNA	Fold Change	FDR *p*-Value	*p*-Value
hsa-let-7c-5p	−5.42	0.03	2.83 × 10^−4^
hsa-miR-200a-3p	−2.77	0.09	1.17 × 10^−3^
hsa-miR-3168	−423.00	0.01	5.06 × 10^−5^

**Table 4:** Down- and upregulated miRNAs in non-responders vs. responders with a *p*-value ≤ 0.05.

miRNA	Fold Change	FDR *p*-Value	*p*-Value
hsa-let-7c-5p	−5.42	0.03	2.83 × 10^−4^
hsa-miR-23b-3p	−2.95	0.83	0.03
hsa-miR-27b-3p	−2.25	0.83	0.03
hsa-miR-141-3p	−2.44	0.55	0.01
hsa-miR-143-3p	1.57	0.81	0.02
hsa-miR-155-5p	−6.41	0.55	0.01
hsa-miR-200a-3p	−2.77	0.09	1.17 × 10^−3^
hsa-miR-3168	−423.00	0.01	5.06 × 10^−5^

**Table 5:** Down- and upregulated miRNAs in cases vs. controls.

miRNA	Fold Change	FDR *p*-Value	*p*-Value
hsa-miR-93-5p	−2.34	0.07	3.09 × 10^−4^
hsa-miR-3168	366.81	3.94 × 10^−3^	9.33 × 10^−6^

**Table 6:** Down- and upregulated miRNAs in patients vs. controls with a *p*-value ≤ 0.05.

miRNA	Fold Change	FDR *p*-Value	*p*-Value
hsa-miR-3168	366.813977	0.003935	0.000009325
hsa-miR-93-5p	−2.335258	0.06522058	0.0003091
hsa-miR-205-5p	−2.4198197	0.3798608	0.0027
hsa-miR-181a-5p	2.16581448	0.41298667	0.004047
hsa-miR-486-5p	−10.228425	0.41298667	0.004893
hsa-miR-451a	−65.679732	0.43645454	0.006206
hsa-miR-320b	21.8041849	0.75253088	0.01248274
hsa-miR-29a-3p	−1.7708513	0.79538712	0.01507843
hsa-miR-19a-3p	2.95052194	0.8252134	0.03392701
hsa-miR-144-5p	−9.8621314	0.8252134	0.04115207
hsa-miR-182-5p	−2.4971916	0.8252134	0.04473483
hsa-miR-145-5p	−16.732144	0.8252134	0.04643798
hsa-miR-1290	1.99031054	0.8252134	0.04793907

**Table 7:** Down- and upregulated miRNAs in responders vs. controls.

miRNA	Fold Change	FDR *p*-Value	*p*-Value
hsa-miR-93-5p	−2.05	0.10	9.52 × 10^−3^
hsa-miR-143-3p	1.60	0.10	8.89 × 10^−3^
hsa-miR-205-5p	−3.50	2.96 × 10^−3^	9.86 × 10^−5^

**Table 8:** MiRNAs that are differentially expressed in this study and reported in the literature as associated with endometriosis (↑ = up-regulated, ↓ = down-regulated).

miRNA	Expression Level	Sample	Literature Reference
hsa-miR-27b-3p	↓	Human endometrial stromal cells (exposed to estradiol)	[57]
	↑	Endometrial stromal cells (endometriosis vs. controls)	[58]
	↑	Human endometriosis cell line hEM15A	[59]
hsa-miR-93-5p	↑	Endometriomas vs. ovarian cancers	[60]
	↓	Superficial endometriosis vs. deep endometriosis/endometriomas	[47]
	↑	Endometrial mesenchymal stromal cells (treated with endometriotic serum)	[61]
	↑	Endometriomas vs. eutopic endometrium	[9]
	↓ (DROSHA downregulation)	In silico analysis, endometriotic mesenchymal stromal cells (MenSCs)	[48]
	↓	Endometriotic lesions	[49]
hsa-miR-200a-3p	↓	Ectopic vs. eutopic endometrium	[62]
hsa-miR-3168	↓	Blood of endometriosis patients vs. controls	[44]
hsa-miR-23b-3p			No Studies
hsa-miR-141-3p			No Studies
hsa-miR-143-3p	↑	Endometriotic stromal cells vs. normal endometrial stromal cells	[53]
	↑	Ectopic vs. eutopic and normal endometrial tissues	[63]
	↑	Endometriosis vs. ovarian endometrioid carcinoma/endometrioid endometrial cancer	[64]
	↑	Serum of endometriosis patients vs. controls	[50]
	↓	Plasma of endometriosis patients vs. controls	[51]
	↓	Eutopic endometrium of women with endometriosis vs. controls	[47]
hsa-miR-155-5p			No Studies
hsa-let-7c-5p			No Studies
hsa-miR-205-5p	↓	Ectopic endometrium of endometriosis patients vs. controls	[65]

## Data Availability

The raw data supporting the conclusions of this article will be made available by the authors on request.

## Abbreviations

The following abbreviations are used in this manuscript:

miRNA	microRNA
PRs	Progesterone Receptors
BMI	Body Mass Index
NRS	Numerical Rating Scale
PGIC	Patient’s Global Impression of Change
EMT	Epithelial-to-Mesenchymal Transition
VEGF	Vascular Endothelial Growth Factor
